# Supplementary material for: Deletion of wfs1 Impairs Oligodendrocyte Precursor Cells Dorsal Distribution and Myelination Through the wfs1-hmgcs1 Axis in Zebrafish
Source: Biology (Basel). 2026 Aug 16;15(16):1402. doi: 10.3390/biology15161402 (PMC13510151; doi:10.3390/biology15161402)
Supplement: Supplementary file 1 [file biology-15-01402-s001.zip › File S1-Transcriptome sequencing data and differentially expressed genes (DEGs) in wfs1-- zebrafish at 5 dpf/2.KEGG/wfs1KOvsWT/UP/src/dre00100.html]

dre00100


- dre:406662

- Up regulated genes

406662\_2(2.74999511466069)

- dre:322481

- Up regulated genes

322481(1.25923363606388)
- dre:565117

- Up regulated genes

565117(2.67130454108397)

- dre:100004700

- Up regulated genes

100004700(1.17582821096165)

- dre:100004700

- Up regulated genes

100004700(1.17582821096165)

- dre:406662

- Up regulated genes

406662\_2(2.74999511466069)

Close
